# Intensification of insulin therapy in patients with type 2 diabetes: a retrospective, non- interventional cohort study of patients treated with insulin glargine or biphasic human insulin in daily clinical practice

**DOI:** 10.1186/1758-5996-5-43

**Published:** 2013-08-05

**Authors:** Nicholas Tentolouris, Venetsana Kyriazopoulou, Dimitrios Makrigiannis, Barbara Baroutsou

**Affiliations:** 1 1st Department of Propaedeutic and Internal Medicine, Athens University Medical School, Laiko General Hospital, 33 Lakonias Street, 115 23, Athens, Greece; 2 Department of Internal Medicine, Division of Endocrinology and Diabetes, Patras University Medical School, University Hospital of Patras, Patras, Greece; 3 Department of Internal Medicine, General Hospital of Ioannina Chatzikosta, Ioannina, Greece; 4 sanofi – aventis, Athens, Greece

**Keywords:** Biphasic human insulin, Insulin glargine, Glycaemic control, Hypoglycaemia, Body weight

## Abstract

**Background:**

The aim of this study is to compare the efficacy of intensification of insulin treatment with insulin glargine and biphasic human insulin in patients with type 2 diabetes on concomitant therapy with oral antidiabetic drugs (OAD) in daily clinical practice.

**Methods:**

A retrospective multicentre parallel two-arm study included 301 patients with type 2 diabetes already on treatment with biphasic human insulin twice daily (bd) in combination with OAD. Data were collected retrospectively from 142 patients who had been switched from biphasic human insulin to insulin glargine in a period of 6–12 months prior to their inclusion (active group) and compared to data collected retrospectively from 159 patients who continued treatment with biphasic human insulin bd for the same time period (control group). Our primary objective was to examine the efficacy of the two treatments, assessed as change in HbA1c. Secondary objectives were to examine for changes in fasting blood glucose (FBG), body weight, treatment with OAD or fast-acting insulin and safety, by assessing the frequency and severity of hypoglycaemic episodes.

**Results:**

At the end of the study there was a significant reduction in HbA1c in both arms. The least squares (LS) mean [(95% confidence intervals (CI)] reduction in HbA1c was -1.13 (-0.96 to -1.30)% in the active and -0.59 (-0.41to -0.77)% in the control group [LS mean treatment difference 0.53 (0.31-0.76)%, p < 0.001]. Similarly, fasting blood glucose declined significantly in both arms. The LS mean decline in FBG was -47.02 (-37.89 to -56.14) mg/dl in the active and -19.73 (-11.57 to -27.89) mg/dl in the control group [LS mean treatment difference 27.85 (15.74-39.95) mg/dl, p < 0.001]. No significant difference in hypoglycaemic episodes and in body weight was found. In the active group, more patients received rapid-acting pre-meal insulin and used insulin secretagogues drugs.

**Conclusions:**

Glargine alone or in combination with fast acting insulin is more effective in reducing glycaemia than biphasic human insulin alone or in combination with fast acting insulin in patients with type 2 diabetes without increase in hypoglycaemic episodes or body weight.

## Introduction

Insulin administration in patients with type 2 diabetes mellitus (T2DM) has proved to be the most effective treatment modality, which should be initiated early in the course of the disease in case lifestyle modification and/or metformin administration fail to achieve recommended glycaemic standards [1,2]. Insulin can be administered in addition to oral hypoglycaemic therapy and in different therapeutic schemes which can be prospectively modified based on close glycosylated haemoglobin A1c (HbA1c) and blood glucose (BG) monitoring [3].

The primary objective of the present retrospective study (LANTus utilisation in real life versus PREmix Insulin, PRELANTI) was to assess the efficacy of intensification of treatment, by measuring the change in HbA1c, of two main insulin treatment schemes: (a) glargine once daily in the evening and (b) biphasic human insulin twice daily (bd) in patients with T2DM on concomitant therapy with oral antidiabetic drugs (OAD). Secondary objectives were to examine for changes in fasting BG, body weight, treatment with OAD or insulin and safety, by assessing the frequency and severity of hypoglycaemic episodes. In both treatment groups, fast acting insulin was added if needed.

## Research design and methods

A retrospective parallel two-arm study was designed to include 332 patients with T2DM, equally distributed in two treatment groups. Taking into consideration that difference in HbA1c change is 0.34% in favour of insulin glargine versus premixed insulins in similar comparative studies between insulin treatment strategies [4], a sample size of 332 participants were required to be enrolled in the study with an alpha risk (2-sided test) of 5% and 80% power, as well as with a standard deviation of 1.1%. Finally, 301 patients, already on treatment with biphasic human insulin bd in combination with OADs, were recruited in 10 centers (including both National Health Services and university hospitals and private practice doctors) throughout Greece. The study was approved by hospitals research ethics committees and by National Organization for Medicines.

Patients’ selection by investigators and inclusion in the study was taking place consecutively, starting from most recent patient’s data (from each patient’s last visit) and going backwards in time. In each centre, data were collected retrospectively from 142 consecutive patients with T2DM, who had been switched from human biphasic insulin to insulin glargine once daily in a period of 6 – 12 months prior to their inclusion in the study (active group), and compared to data collected retrospectively from 159 consecutive patients who continued treatment with biphasic human insulin bd for the same time period of 6 – 12 months prior to their inclusion in the study (control group). Fast acting insulin was added in both groups if considered necessary, depending on the values of HbA1c and BG at the attending physician’s discretion. OADs were also added or withdrawn on the basis of the attending physician’s global assessment of the efficacy of treatment schemes and the changes were reported on the patients’ case records. Baseline characteristics of study population are given in Table 1.

**Table 1:** Baseline characteristics of the study population

**Characteristic**	**Control group (n = 159)**	**Active group (n = 142)**	**p-value**
**Sex, n (%)**			
**Male**	73 (45.9)	60 (42.3)	0.523
**Female**	86 (54.1)	82 (57.8)	
**Age (years)**	66.7 ± 9.6	63.2 ± 10.1	0.002
**BMI (kg/m**^**2**^**)**	31.1 ± 8.6	29.3 ± 5.4	0.029
**Height (cm)**	162.8 ± 12.5	164.6 ± 8.4	0.146
**Weight (kg)**	80.8 ± 12.7	79.3 ± 14.2	0.331
**Years diagnosed**	16.0 ± 8.6	13.5 ± 7.8	0.011
**Duration of monitoring period (months)**	9.3 ± 1.9	9.1 ± 2.0	0.291
**HbA1c (%)**	8.18 ± 1.33	8.53 ± 1.29	0.019
**FBG (mg/dl)**	168 ± 47.2	182.6 ± 57.1	0.025

Data are mean ± SD or n (%).

Data were collected from the medical records for all studied variables and for total insulin dose administered, number of injections and changes in body weight during the monitoring period. Regarding hypoglycaemia, we reported the number and the severity of hypoglycaemic episodes as well nocturnal hypoglycaemia. Severe hypoglycaemia was defined as any hypoglycaemic episode that the patient was unable to self-treat requiring the assistance of another person to deal with it [5]. All the other cases of hypoglycaemia (documented symptomatic hypoglycaemia, asymptomatic hypoglycaemia, probable symptomatic hypoglycaemia and relative hypoglycaemia) were considered as mild/moderate hypoglycaemia [5].

### Statistical analysis

Data were analyzed separately in each treatment group (control/active group). Continuous variables were presented by measures of central tendency and dispersion, whereas categorical variables by frequency distribution tables. The comparability of the two treatment groups regarding baseline data was assessed by means of t-student distribution tests for continuous variables and via chi-square Pearson’s tests of independence or Fisher’s exact tests for categorical variables, respectively. The least squares (LS) mean differences and two-sided 95% confidence intervals (CI) were estimated for the comparisons of the changes in HbA1c, FBG and body weight between the active and the control group.The comparability of treatment groups regarding the number of hypoglycaemic incidences was assessed by means of Wilcoxon rank-sum test. The evaluation of the change in the proportion of patients receiving orally administered antidiabetic treatment from the start until the end of the monitoring period was performed by means of McNemar test. The statistically significant variables were identified according to the analysis of covariance (ANCOVA) methodology. The statistical significance of body weight changes in each treatment group was evaluated by one-way analysis of variance (ANOVA) tests. The hypothesis of equal hypoglycaemic incidence rates between the two treatment groups was assessed by the estimation of relative risk (RR) and 95% confidence intervals (CI), while the hypothesis of equal rapid acting insulin intake was assessed by the estimation of odds ratio (OR, 95% CI). All statistical tests were two-tailed and the significance level was set to 5%.

## Results

### Baseline characteristics

At baseline, the patients of the active group were younger compared to the control group and this difference reached statistical significance. Furthermore active group had a significantly lower body mass index and shorter known duration of diabetes mellitus. In addition, active group had a significantly higher HbA1c and fasting BG when compared to control group (Table 1).

Equal number of participants had been treated with metformin, sulfonylurea, meglitinides and alpha-glucosidase inhibitors in the control and the active group at baseline. However, more patients received thiazolidinediones in the active group (Table 2).

**Table 2:** Orally administered anti-diabetic drugs (OAD) and insulin at baseline and the end of the monitoring period

	**Control group**	**Active group**	**p-value**
**Start of the monitoring period n (%) or mean value ± SD**			
Metformin	71 (44.7)	64 (45.1)	0.942
Sulfonylureas	15 (9.4)	14 (9.9)	0.901
Meglitinides (repaglinide/nateglinide)	0 (0.0)	0 (0.0)	-
Thiazolidinedions	3 (1.9)	10 (7.0)	0.028
Alpha-glucosidase inhibitor	2 (1.3)	1 (0.7)	1.0
Other*	1 (0.6)	0 (0.0)	1.0
Biphasic human insulin (U/day)	47.84 ± 20.05	44.75 ± 19.85	0.18
Biphasic human insulin (U/kg/day)	0.61 ± 0.27	0.56 ± 0.25	0.13
**End of the monitoring period n (%)**			
Metformin	63(39.6)	62 (43.7)	0.478
Sulfonylurea	2 (1.3)	18 (12.7)	<0.001
Meglitinides (repaglinide/nateglinide)	0 (0.0)	6 (4.2)	0.009
Thiazolidinedions	1 (0.6)	7 (4.9)	0.021
Alpha-glucosidase inhibitor	1 (0.6)	1 (0.7)	1.0
Other*	1 (0.6)	2 (1.4)	0.604
Total insulin (glargine or biphasic plus prandial) dose (U/day)	50.89 ± 21.56	42.97 ± 21.53	<0.001
Total insulin (glargine or biphasic plus prandial) dose (U/kg/day)	0.65 ± 0.28	0.53 ± 0.26	<0.001
**Change [%, 95% confidence intervals, (CI)] in the OAD and daily insulin dose between baseline and the end of the monitoring period**			
	%, 95% CI	%, 95% CI	
Metformin	-5.0 (-9.5, -0.4)	-1.4 (-6.6, 3.8)	
p-value (baseline vs end)	0.033	0.593	
Sulfonylureas	-8.1 (-12.9, 0.4)	2.8 (-3.9, 3.8)	
p-value (baseline vs end)	0.001	0.393	
Meglitinides (repaglinide/nateglinide)	0.0	4.2 (0.8, 7.6)	
p-value (baseline vs end)	-	0.014	
Thiazolinidiones	-1.3 (-3.0, 0.5)	-2.1 (-5.2, 1.0)	
p-value (baseline vs end)	0.157	0.180	
Alpha-glusidase inhibitor	0.0	0.0	
p-value (baseline vs end)	0.317	-	
Other*	0.0 (-1.7, 1.7)	1.4 (-0.5, 3.4)	
p-value (baseline vs end)	1.0	0.157	
Total insulin (glargine or biphasic plus prandial) dose (U/day)	3.49 (2.16, 4.82)	-1.78 (-5.25, -1.69)	
p-value (baseline vs end)	<0.001	0.01	
Total insulin (glargine or biphasic plus prandial) dose (U/kg/day)	0.04 (0.02, 0.06)	-0.02 (-0.07, -0.01)	
p-value (baseline vs end)	<0.001	0.007	

*Other includes dipeptidyl-peptidase 4 inhibitors or glucagon-like peptide 1 mimetics.

### Efficacy of treatment

**Figure 1:**
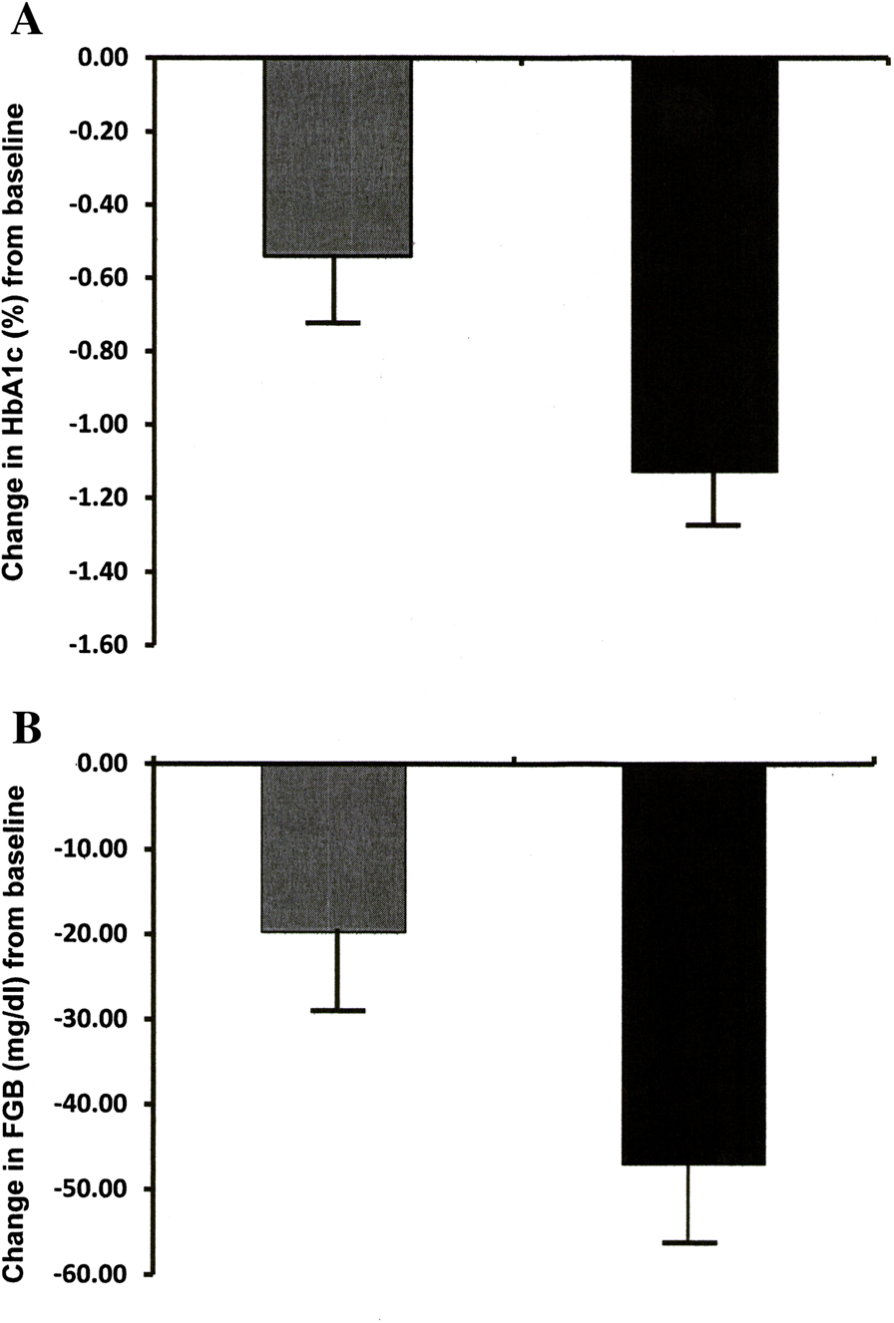
**Changes in HBA1c and fasting blood glucose levels. A** The reduction in HbA1c levels in the control (grey bars) and the active group (black bar) after 6–12 months of follow up. Least squares mean difference between the active and the control group 0.53 (0.31-0.76)%, p < 0.001. **B** The reduction in fasting blood glucose levels (FBG) in the control (grey bars) and the active group (black bar) after 6–12 months of follow up. Least squares mean difference between the active and the control group 27.85 (15.74-39.95) mg/dl, p < 0.001.

During the monitoring period there was a significant reduction in HbA1c levels in both treatment groups. The LS mean (95% CI) reduction of HbA1c value was -1.13 (-0.96 to -1.30)%] in the active and-0.59 (-0.41 to -0.77)%] in the control group. The LS mean (95% CI) difference of HbA1c between the active and the control group was 0.53 (0.31-0.76)% (p < 0.001) (Table 3, Figure 1A). At the end of the monitoring period a total of 29.6% of patients in the active and 23.2% of patients in the control group reached HbA1c < 7% (χ^2^ = 1.54, p = 0.21) and 6.3% of patients in the active and 9.4% of patients in the control group reached HbA1c < 6.5% (χ^2^ = 0.64, p = 0.42).

**Table 3:** HbA1c and fasting blood glucose (FBG) levels per treatment group at baseline and the end of the monitoring period

	**Control group**	**Active group**	**p-value†**
**HbA1c (%)**			
Prior treatment	8.18 ± 1.33	8.53 ± 1.29	
After treatment	7.58 ± 1.06	7.39 ± 0.81	
p-value (baseline vs end)‡	<0.001	<0.001	
**LS mean difference (95% CI)**	0.53 (0.31-0.76)		<0.001
**FBG (mg/dl)**			
Prior treatment	168.9 ± 47.2	182.6 ± 57.1	
After treatment	148.5 ± 42.7	135.6 ± 34.5	
p-value(baseline vs end) ‡	<0.001	<0.001	
**LS mean difference (95% CI)**	27.85 (15.74-39.95)		<0.001

^†^Statistically significant difference between the control and the active group.

^‡^Statistically significant difference in least squares means of HbA1c in each treatment group.

*CI* confidence intervals.

*LS* least squares.

Fasting BG declined significantly in both treatment groups during the monitoring period. The LS mean decline in FBG was -47.02 (-37.89 to -56.14) mg/dl in the active and -19.73 (-11.57 to -27.89) mg/dl in the control group The LS mean (95% CI) difference of fasting BG between the active and the control group was 27.85 (15.74-39.95) mg/dl (p < 0.001) (Table 3, Figure 1B).

### Hypoglycaemic episodes

The monthly number of mild/moderate hypoglycaemic episodes, the nocturnal episodes, and the serious hypoglycaemic episodes did not differ significantly between the treatment groups during the monitoring period (Tables 4 and 5).

**Table 4:** Monthly number (mean ± SD) of hypoglycemic episodes and number (%) of patients with hypoglycaemia per treatment group during the reporting period of treatment with biphasic insulin or insulin glargine

**Hypoglycemic episodes per month**	**Control group**	**Active group**	**p-value**
**Mild/moderate**	0.757 ± 2.294	0.705 ± 1.852	0.926
**Nocturnal**	0.076 ± 0.466	0.053 ± 0.195	0.961
**Serious**	0.017 ± 0.182	0.007 ± 0.063	0.833

**Table 5:** Relative risk (RR) for at least one hypoglycaemic episode occurrence between patients of both treatment groups

**Hypoglycaemic episodes**	**Control Group**	**Active Group**	**RR (95% confidence intervals)**	**p-value**
**Mild/moderate**	58 (38.7)	56 (39.7)	0.98 (0.82, 1.18)	0.855
**Nocturmal**	20 (13.4)	18 (12.8)	1.01 (0.92, 1.10)	0.869
**Serious**	3 (2.0)	3 (2.1)	1.00 (0.97, 1.03)	1.0

### Changes in body weight

In the control group, body weight at baseline was 80.8 ± 12.7 kg and at the end of the monitoring period 81.6 ± 12.9 kg (p = 0.331); the LS mean (95% CI)% change in body weight was 0.734 (0.139, 1.330) (p = 0.012). In the active group, body weight was 79.3 ± 14.2 kg at baseline and 79.4 ± 14.4 kg at the end of the monitoring period (p = 0.170).; the LS mean (95% CI)% change in body weight was -0.121 (-0.754, 0.511) kg. The LS mean (95% CI)% difference in body weight change between the active and the control group was 0.856 (-0.013, 1.725) kg (p = 0.054).

### Daily insulin use

At baseline, the daily dose of biphasic human insulin was not different between the active and the control group (p = 0.18). At the end of the monitoring period, in the control treatment group there was an increase in the total daily dose of insulin in comparison with the baseline (from 47.84.0 U/day to 50.89 U/day, p < 0.001). At the end of the monitoring period, in the active treatment group, there was a reduction in the total daily dose of insulin (from 44.75 U/day to 42.97 U/day, p < 0.001). Similar changes were found in total daily insulin dose expressed in U/kg/day (Table 2).

At the end of the monitoring period, a total of 13 patients in the control group (8.2%) and 99 patients in the active group (69.7%) received pre-meal rapid acting insulin [OR: 0.04; 95% CI (0.02, 0.08), p < 0.001]. The percentage of the patients who received rapid-acting insulin in the active group was by 58.7% (95% CI: 49.2, 68.2; p < 0.001) higher than that in the control group. The mean value of the rapid-acting insulin units received by the patients in the active group was by 13.7U/day (95% CI: 3.6, 23.9; p < 0.001) greater than that received by patients of the control group. The number of insulin injections and the dose of the rapid-acting insulin at the end of the monitoring period in the two treatment groups is shown in Table 6.

**Table 6:** Number of daily rapid-acting insulin injections, biphasic or glargine insulin and total daily insulin dose after the end of the monitoring period

	**Control group**	**Active group**	**p-value**
**Number of insulin injections**, **n (%)**			
1	16 (10.1)	45 (31.7)	
2	85 (53.5)	20 (14.1)	
3	58 (36.5)	27 (19.0)	<0.001
4	0 (0.0)	49 (34.5)	
5	0 (0.0)	1 (0.7)	
**Mean** ± **SD daily units of rapid acting insulin (U/day)**	9.5 ± 2.3	23.3 ± 18.4	<0.001
**Mean** ± **SD daily units of rapid acting insulin (U/Kg/day)**	0.12 ± 0.03	0.29 ± 0.23	<0.001

At the end of the study, a total of 31.7% of the patients in the active group were managed only with basal insulin glargine, while the rest of the patients needed the addition of prandial insulin (one dose: 14.1%; two doses: 19.0%; three: doses: 34.5%; and four doses: 0.7%). A total of 10.1% of the patients in the control group were managed with one injection of biphasic insulin, 53.5% with 2 injections daily and 36.5% of the patients with 3 injections daily (Table 6).

### Concomitant treatment with OAD

Metformin was discontinued in 5% (p = 0.003) of the patients in the control and in 1.4% of the patients in the active group (p = 0.593) by the end of the study period. In addition, the use of sulfonylureas was reduced by 8.1% (p = 0.001) in the control and was increased by 2.8% (p = 0.393) in the active group. A significant also increase (by 4.2%, p = 0.014) was found in the use of meglitinides in the active group by the end of the monitoring period. No other significant change in the use of other OAD was noticed during the follow up period (Table 2).

## Discussion

The importance of good glycaemic control constitutes the cornerstone of every therapeutic modality in patients with diabetes. Prospective randomised studies have clearly shown that effective diabetes control, as judged by HbA1c, is associated with lower incidence of chronic complications, especially microangiopathy, both in patients with type 1 and type 2 diabetes [6,7]. The same applies to satisfactory targeting of postprandial hyperglycaemia that constitutes an independent risk factor for the development of diabetic complications [8].

The therapeutic goals in terms of HbA1c level s and of fasting and post prandial blood glucose values have been based on large epidemiological studies associating glucose control with the incidence of diabetic complications, although the therapeutic target and timing of intervention for the prevention of macroangiopathic complications have not been well documented compared to relevant action for the reduction of microangiopathy complications [9-11].

The need for effective glycaemic control in the early stages of the disease has modified treatment of T2DM in patients not adequately controlled with life style changes and oral hypoglycaemic agents, bringing insulin into prominence as an effective therapeutic intervention even in the early stages of the disease and before secretory failure of β-cells occurs. The combination of oral hypoglycaemic agents and insulin administration has become a favourable type of treatment achieving effective control with lower insulin dosage in T2DM. The benefits of insulin administration on preservation of β-cell secretory capacity, the antinflammatory action and the dose dependent hypoglycaemic activity are combined with the favourable effects of some OAD like metformin on insulin resistance and glucagon-like-peptide-1 (GLP-1) agonists and dipeptyl-peptidase-4 (DPP-4) inhibitors on β-cell mass preservation and appetite as well as on weight control [12,13].

The advent of insulin analogues and especially the basal insulins have facilitated initiation of insulin treatment and reduced the incidence of hypoglycaemic episodes which was a certain barrier for insulin therapy in the early stages of T2DM [14]. The treatment guidelines suggested by the American Diabetes Association (ADA) and the European Association for the Study of Diabetes (EASD) [1,2,15] and the introduction of simple instructions for insulin titration [14-18] resulted in an increase of the use of insulin in patients with T2DM.

Insulin may be given in different therapeutic schemes either alone or in combination with OAD depending on the severity of the disease and appointed glycaemic targets. It can be administered once daily as basal insulin usually in combination with 1 or 2 OAD, basal insulin in combination with fast acting insulin, premeal bolus short acting insulin or in the form of a premixed intermediate acting/fast acting combinations (biphasic) insulin [15].

In the present study we assessed the efficacy and safety of two insulin regimens in subjects with T2DM who had been already on treatment with biphasic human insulin in combination with OAD, without achieving optimal glycaemic control.

A total of 142 patients who had switched treatment from biphasic human insulin to insulin glargine daily were included in the active group and compared to 159 patients who continued treatment with biphasic human insulin bd and were included in the control group. We have shown that the reduction in HbA1c and fasting BG values achieved were significantly greater in the active than in the control group. In particular, HbA1c declined by 1.13% in the active and by 0.59% in the control group and fasting BG declined by 47 mg/dl in the active and 20 mg/dl in the control group.

It must also be emphasized that despite more intensive management in the active group, the number of hypoglycaemic episodes did not differ and body weight did not increase in comparison with the control group. Moreover, only one third of the patients in the active treatment group required three injections of fast acting insulin in addition to basal insulin glargine, the dosage of which was considerably reduced at the end of the monitoring period as a consequence of the addition of fast acting insulin.

Well controlled studies on the comparative evaluation of the effectiveness and safety of the different insulin schemes are limited. Raskin et al. [19] in a 28-week parallel group randomized study involving 233 insulin-naïve patients with T2DM on treatment with metformin, found that biphasic insulin twice daily was more effective in lowering HbA1c compared to treatment with once daily insulin glargine. However, weight gain was greater and minor hypoglycaemic episodes were more often in the group treated with biphasic insulin. Similar results were presented by Holman et al. [17] in an open label one year controlled study involving 708 patients on metformin and sulfonylurea treatment randomized in three insulin treatment groups, biphasic insulin twice daily, fast acting insulin three times daily or basal insulin once daily (or twice if required). However, their results were modified three years after randomization and patients who added a basal or fast acting insulin-based regimen had better glycaemic control than patients who added biphasic insulin [20].

Another study examined the efficacy and safety of two treatment regimens (intensified basal-bolus glargine/glulisine regimen and biphasic insulin twice daily) for 52 weeks in 312 patients with long-standing T2DM initially treated with biphasic insulin. The study showed that patients allocated in the basal-bolus regimen had superior glycaemic control vs. those allocated in the premix therapy with no increase in the rates of hypoglycaemia [21]. In addition more patients reached HbA1c ≤ 7% in the basal-bolus than in the biphasic treatment group (46.6% vs. 27.9%).

In a meta-analysis, Lasserson et al. [22] reviewed 22 trials that randomized 4,379 insulin-naïve patients. They found greater HbA1c reductions with biphasic and fast acting insulin compared with basal insulin, but at the end of the study larger doses were needed in the biphasic and fast acting arms with minor hypoglycaemic events being inconsistently higher in biphasic and fast acting groups and weight gain greater in the fast acting group compared to the group on basal insulin.

In our study the addition of fast acting insulin when necessary to basal insulin glargine resulted in greater HbA1c and fasting BG reduction, which almost reached the suggested by the ADA/EASD guidelines levels [1,2,15]. This was achieved with no increase in weight or in the rate of hypoglycaemic episodes. The recent guidelines for the management of T2DM suggest that not all patients need or benefit from aggressive glucose management and that it is important to individualize treatment targets [15]. Because most of the T2DM patients maintain some endogenous insulin secretion even in late stages of disease, addition of basal insulin [neutral protamine Hagedorn (NPH) or long-acting insulin glargine or insulin detemir)] to the OAD if glucose control is inadequate is an effective, safe and simple approach, unless the patient is markedly hyperglycemic [15]. Our results showed that almost 30% of the participants managed to achieve glucose targets only with insulin glargine once daily. However, almost 40% of the participants in the control and 54% of the participants in the active group needed 3or more insulin injections per day. Moreover, we found that with basal insulin regimens, the addition of medications-beyond metformin-that increase insulin secretion like sulfonylureas or meglitinides is necessary in some patients for the maintenance of glycaemic control; thus, more patients in the active group received such medications during follow-up. Therefore, our data suggest that addition of basal insulin to OAD is an effective approach for the management of hyperglycaemia in T2DM and intensification of insulin therapy with the addition of fast-acting pre-meal insulin may be necessary to achieve glucose targets.

The combination of basal insulin with fast acting insulin when needed offers greater flexibility and better glycaemic control mimicking the physiologic insulin secretion. Due to its pharmacodynamic profile, insulin glargine substitutes basal insulin secretion effectively controlling morning glycaemia, whereas fast acting insulin reduces postprandial hyperglycaemia.

The strength of this study is that it examined in everyday clinical practice with no intervention at all and with adequate power the effectiveness of intensification of treatment with insulin based on two insulin regimens (basal insulin glargine vs. biphasic human insulin) on glycemic control. In addition, the study has adequate power to support the findings. However, this study being observational and retrospective by design has several limitations. First, the two groups differ significantly at baseline in terms of age, BMI, known duration of diabetes and diabetes control. Moreover, at the end of the monitoring period there were significant differences in concomitant treatments for diabetes; thus the results should be interpreted taking into consideration the changes in medications used for the treatment of diabetes during the study. Second, the number of mild/moderate hypoglycaemic episodes might have been under-reported in medical records and therefore, underestimated. Third, detailed data for documented symptomatic hypoglycaemia, asymptomatic hypoglycaemia, probable symptomatic hypoglycaemia and relative hypoglycaemia were not available and all cases of hypoglycaemia other than severe hypoglycaemia were classified as mild or moderate hypoglycaemia. Fourth, causality between the treatments and the outcomes cannot be established and these findings must be confirmed by a randomized clinical trial.

## Conclusions

Our findings suggest that treatment with basal insulin glargine alone or in combination with fast acting insulin is more effective in reducing glycaemia compared to treatment with biphasic human insulin alone or in combination with fast acting insulin in patients with T2DM already on treatment with biphasic human insulin only, without any increase in hypoglycaemic episodes or body weight.

## Competing interests

Nicholas Tentolouris has received honoraria from NovoNordisk, Sanofi, Elli-Lilly, Bristol-Myers-Squibb and Novartis for memberships on advisory boards and for speaking on subjects related to treatment with antidiabetic drugs and insulin. Venetsana Kyriazopoulou declares no conflict of interest. Dimitrios Makrigiannis has received honoraria from NovoNordisk, Sanofi, Elli-Lilly and Novartis for memberships on the advisory boards and for speaking on subjects related to treatment with antidiabetic drugs and insulin. Barbara Baroutsou is an employee of Sanofi working as Medical Director for Greece and Cyprus.

## Authors’ contributions

NT was principal investigator, study co-ordinator and investigator, participated in all stages of recruitment of the patients and in analysis of the data, drafted and reviewed critically the manuscript. VK was study co-ordinator and investigator, participated in all stages of recruitment of the patients and in analysis of the data, drafted and reviewed critically the manuscript; DM was study investigator, participated in all stages of recruitment of patients and reviewed critically the manuscript; BB was study sponsor co-ordinator, wrote the protocol, drafted and reviewed critically the manuscript. All other study investigators conducted the study and collected the data. All authors read and approved the final manuscript.

## Authors’ information

Study investigators

N. Tentolouris and I. Mpalla, 1st Department of Propaedeutic and Internal Medicine, Athens University Medical School, Laiko General Hospital, Athens; V. Kyriazopoulou and M. Michalaki, Department of Internal Medicine, Division of Endocrinology and Diabetes, University Hospital of Patras, Patras; I. Ioannidis, 2nd Department of Internal Medicine, Konstantopoulio Hospital, Nea Ionia; E. Pagkalos, 1st Department of Internal Medicine, Papageorgiou Hospital, Thessaloniki; C. Vasilopoulos Department of Endocrinology, General Hospital of Athens “Evangelismos”, Athens; V. Klisiaris, Department of Internal Medicine, General Hospital of Larisa “Koutllibanio-Triantafilio”, Larisa; D. Makrigiannis, Department of Internal Medicine, General Hospital of Ioannina “Hatzikosta”, Ioannina; I. Mallias, Second Department of Internal Medicine General Hospital of Serres, Serres; K. Kazakos, Internist, Diabetologist, Thessaloniki; A. Likoudi Internist - Diabetologist, Marathonas, Athens.
